# School travel experiences of learners in rural areas: the case of Mt Elias, KwaZulu-Natal

**DOI:** 10.3389/fsoc.2026.1751964

**Published:** 2026-03-11

**Authors:** Babra Duri, Blessing Takawira

**Affiliations:** Institute of Transport and Logistics Studies (Africa), University of Johannesburg, Johannesburg, South Africa

**Keywords:** education, infrastructure, learners, school access, transport

## Abstract

**Introduction:**

In South Africa, the right to basic education is constitutionally protected; however, learners in geographically marginalized communities continue to face severe mobility constraints that undermine school attendance, safety, and engagement. The purpose of the study was to understand the daily school travel experiences of learners in the rural community of Mt. Elias, KwaZulu-Natal.

**Methods:**

A qualitative, exploratory design was used to address the research questions. Twenty-three Grade 6 and 7 learners from Mt Elias participated in this study. Mt Elias is a rural area characterized by dispersed settlements, steep valleys, and limited transport infrastructure.

**Results:**

Learners predominantly walk all the way to and from school. While some learners live nearby schools, those who live deep in the valley of Mt. Elias walk long distances, often over an hour and frequently starting their journeys before dawn. Transport options for commuting to and from school were limited. Environmental hazards included bushy paths, isolated routes, graveyards, animals, and muddy or slippery roads during the rainy season. These conditions contributed to fatigue, lateness, absenteeism, and reduced school engagement.

**Discussion:**

The challenges identified by learners are multifaceted, involving physical, economic, environmental, and emotional hardships. From a transport equity perspective, the study highlighted critical issues of distributive injustice in the uneven allocation of transport services; recognitional injustice in the limited attention to local realities and gendered risks; and procedural injustice in the exclusion of learners and caregivers from transport planning. Addressing these layered inequalities requires not only infrastructure investment, but also responsive, participatory, and context-sensitive policies that center the voices of rural communities.

## 1 Introduction

Education is recognized worldwide as a fundamental human right, established in various national and international legal frameworks. In South Africa, the right to education is explicitly guaranteed in Section 29 of the Constitution of the Republic of South Africa, which asserts that “everyone has the right to a basic education, including adult basic education” (Republic of South Africa, 1996). However, the realization of this right requires supportive structural and infrastructural frameworks, among which access to safe and reliable transport plays a critical role. In rural communities, particularly in geographically marginalized areas, the right to education is often compromised not by pedagogical

limitations but by the severe inadequacies in school transport infrastructure (Department of Transport, 2015; Solminic and Carpenter, 2017).

Equitable education is grounded in the ability of children to physically access learning institutions. In rural communities, where distances between households and schools are often vast and the terrain challenging, transport becomes not merely a convenience but a necessity for learning. The National Learner Transport Policy 2015 emphasizes identifying scholar transport as a critical component of education provision, especially for disadvantaged learners residing in remote areas (Department of Transport, 2015). Yet, many South African rural communities continue to grapple with systemic barriers that undermine this policy's implementation, resulting in many issues such as chronic absenteeism, fatigue, safety risks, and, ultimately, reduced educational outcomes (Manyaka and Mathebula, 2020). In the South African context, the term “scholar transport” encompasses both formal government-subsidized learner transport programmes and privately arranged transport services.

Globally, research highlights the connection between transport equity and education access, particularly in low- and middle-income countries (Anselmo et al., 2020; Hofer-Fischanger et al., 2023). In the context of South African, research shows that rural learners often walk long distances, frequently exceeding 5 kilometers, under physically and emotionally challenging conditions (Porter et al., 2011; Ngidi and Essack, 2022). School journeys in rural communities often include navigating bushy, muddy paths, crossing rivers or busy roads, and passing through socially unsafe areas such as graveyards or informal settlements (Muthige, 2023; Duri et al., 2025). The absence of dedicated school buses, pedestrian infrastructure, and public transport systems exposes learners to significant environmental and social risks during their commute (Ding et al., 2023).

In some parts of South Africa, challenges related to access to educational facilities are exacerbated by mountainous topography and dispersed settlement patterns, where learners often reside in valleys that are distant from educational facilities. Similar patterns have been observed in other rural parts of Africa, where mobility of young people is constrained by long distances to school, structural poverty, poor road conditions, and a lack of institutional support (Porter, 2010; Muthige, 2023; Sibusiso et al., 2025). Barriers to accessing educational facilities disproportionately impact girls and other vulnerable learners, reinforcing and deepening existing inequalities linked to gender, socioeconomic status, and geography. Learners who do not have access to school transport often arrive late, tired, or not at all, particularly during the rainy season when roads become impassable and bridges are flooded (Muthige, 2023; Sibusiso et al., 2025). Academic achievement, student morale, and the perceived worth of education in general may all be impacted by these disturbances. The urban-rural gap in educational performance is wide, undermining the efforts of educators and policy makers to enhance rural education.

Although previous research (Manyaka and Mathebula, 2020; Muthige, 2023; Van Eck et al., 2023) has examined learner transport in various rural contexts across South Africa, it is essential to acknowledge that rural communities are not homogenous. Terrain, climatic conditions, settlement patterns, and the implementation of scholar transport policy differ significantly from one locality to another. Some rural areas benefit from government-subsidized learner transport programmes. However, others remain entirely excluded due to administrative thresholds. These thresholds include rigid distance-based eligibility criteria and fragmented inter-departmental mandates. Additionally, limited financial resources at the provincial level result in fragmented service delivery. At the household level, this financial strain makes private transport unaffordable for those reliant on social grants. Such exclusion reflects a failure of distributive justice, as the benefits of formal transport systems do not reach the learners who require them most. Furthermore, certain regions experience more intense seasonal rainfall, making routes impassable and exposing learners to heightened environmental hazards (Ngidi and Essack, 2022; Muthige, 2023). The spatial dispersion of households in mountainous or valley-bound areas, such as those found in Mt Elias, KwaZulu-Natal presents unique barriers that are not adequately captured in more generalized rural studies.

The primary research question guiding this study is: What are the daily school travel experiences of learners in the rural community of Mt. Elias, KwaZulu-Natal? To address the main question of this study, the study focuses on two key sub-questions, which are (1) How do learners travel to and from school in Mt Elias? (2) What challenges do learners face during their daily travel to school in Mt Elias?

Therefore, this research offers a context-specific contribution that addresses the micro-level variations within rural schooling environments and highlights how localized geography and governance shape educational access and equity. By identifying the mobility challenges faced by learners in Mt Elias, the study contributes to future policy development, such as increasing the availability of subsidized learner transport, improving infrastructure, and implementing safety measures. The study also supports a number of the Sustainable Development Goals (SDGs) of the UN, especially SDG 4 (Quality Education), which aims to promote inclusive and equal access to education by identifying structural and logistical constraints. By highlighting the fact that access to education is only completely achieved when it is ensured, secure, and equal, this study advances our knowledge of the relationship between education, transportation, and rural development.

## 2 Literature review

### 2.1 Theoretical framework

This research is based on the theoretical framework of transport equity. Although there is no universally accepted definition of transport equity (Brown, 2022), it emphasizes the fair distribution of mobility's benefits and burdens to ensure transport systems enable access to vital life opportunities such as education (Pereira and Karner, 2021). There are two main categories of moral issues frequently discussed in the transport equity literature that arise from sufficientarianism and egalitarianism (Pereira and Karner, 2021). Sufficientarianism is a philosophical approach advocating that individuals should have enough resources to meet basic needs and achieve a decent standard of living (Pereira and Karner, 2021). In transport equity, this means ensuring everyone, particularly those from disadvantaged backgrounds, has sufficient access to transport options (Litman, 2018). In the context of this study, sufficientarian perspectives focus on ensuring that all learners can physically reach their schools. Egalitarianism is a philosophical principle advocating for equal treatment and equal access for all individuals, regardless of their background or circumstances (Litman, 2018). Egalitarianism emphasizes that everyone should have the same opportunities to access transport services and facilities (Pereira and Karner, 2021). Egalitarianism in rural school transport emphasizes that all learners, regardless of their geographic location or socio-economic status, should have equal access to safe and reliable transport to their schools. Egalitarianism addresses the unique challenges faced by rural learner who may be at a disadvantage compared urban learners. The principles of equity span three critical dimensions of justice: distributive, recognitional, and procedural justice (Litman, 2018). Distributive justice includes allocating sufficient funding for scholar transport services, ensuring that routes serve remote or underserved areas, and addressing disparities in transport availability between rural and urban learners. Recognitional justice acknowledges the unique challenges rural learners face, such as long travel distances, limited transport options, and safety concerns. Procedural justice in rural school transport involves ensuring that communities have a voice in the planning and decision-making processes related to school transport.

The study also employs a mobility justice theory, which extends the framework by addressing the structural causes of inequality, such as historical spatial dispossession and uneven infrastructure (Bierbaum et al., 2021). Mobility justice effectively ties transport and education equity together by examining who bears the heaviest burdens, such as walking long distances on unsafe roads or facing seasonal and gendered risks, and whose voices are represented in remedial policies (Bierbaum et al., 2021; Flom et al., 2023). Mobility justice provides the analytical tools to examine the uneven exposure to danger and the systemic barriers that define the daily school journeys of rural learners.

### 2.2 Global perspective on school travel in rural areas

Globally, many rural school trips are characterized by long distances, sparse and deteriorated pedestrian infrastructure, limited formal public transport, and elevated exposure to traffic and environmental risk (González et al., 2020; Tengecha et al., 2022). Where learners predominantly walk or cycle, distance and perceived danger are robust correlates of mode choice and of participation in active school transport, often with sharper constraints for lower-income households (González et al., 2020). Safety interventions around schools, crossings, calming, and adult supervision can reduce pedestrian injury, but their effectiveness depends on sustained implementation and local buy-in (Osuret et al., 2024). As a behavioral determinant, perceived safety influences not just the objective crash environment but also the routes and modes that children and caregivers choose based on their perceptions of risk. Travel is made more difficult by seasonal factors, since moderate-to-vigorous physical activity decrease during cold or rain weather, which may have an impact on attendance and welfare (Larouche et al., 2019). Risk in school travel is socially stratified where the disadvantaged learners endure the longest, least resourced routes, face hazardous traffic conditions, and have minimal transport alternatives. Mobility justice therefore, argues for interventions that integrate infrastructure, enforcement, education, and participatory design, attuned to the lived perceptions of children and caregivers (Bierbaum et al., 2021).

Across global and South African literatures, several recurring themes are evident. First, walking predominance and informal transport reliance in rural communities expose learners to compounded risks from distance, infrastructure deficits, and unroadworthy vehicles (Mokitimi and Vanderschuren, 2017). Second, safety and infrastructural challenges, such as the absence of sidewalks, unsafe crossings, speeding, poor lighting, and seasonal hazards, are well-documented and strongly associated with injury and fear (Dimaggio and Li, 2013). Third, gendered and socio-cultural dimensions shape exposure and coping, where female learners face distinct forms of harassment and a pervasive sense of fear when navigating isolated paths or bushy areas, particularly during early-morning commutes. This harassment, occurring within dangerous social geographies, directly informs the gendered risks introduced earlier in this study. From a recognitional justice standpoint, these risks demonstrate how the intersection of geography and gender creates unique gender-based vulnerabilities that necessitate gender-sensitive planning to ensure equitable access to education (Bhana et al., 2021). Fourth, policy-practice disjuncture persist whereby despite national commitments, implementation gaps, fragmented mandates, and governance weaknesses impede equitable provision of scholar transport and safe non-motorized transport (Ally et al., 2024). Lastly, data gaps endure at the local level; accidents, exposure, and mode-share datasets exist for specific rural communities, constraining targeted interventions; seasonal effects and disability inclusion are also under-measured (Gibberd and Hankwebe, 2022).

## 3 Method

### 3.1 Study site

The research was conducted in Mt Elias, a rural community located in the KwaZulu-Natal province of South Africa. Mt Elias is not recognized as a discrete statistical unit in national census or administrative datasets. As a result, disaggregated demographic and socioeconomic data specific to the settlement are not available through Statistics South Africa. Instead, Mt Elias is administratively grouped within broader municipal-level categories. This statistical aggregation reflects a common challenge in rural research, where small and geographically dispersed communities are rendered analytically invisible within broader administrative boundaries.

**Figure 1:**
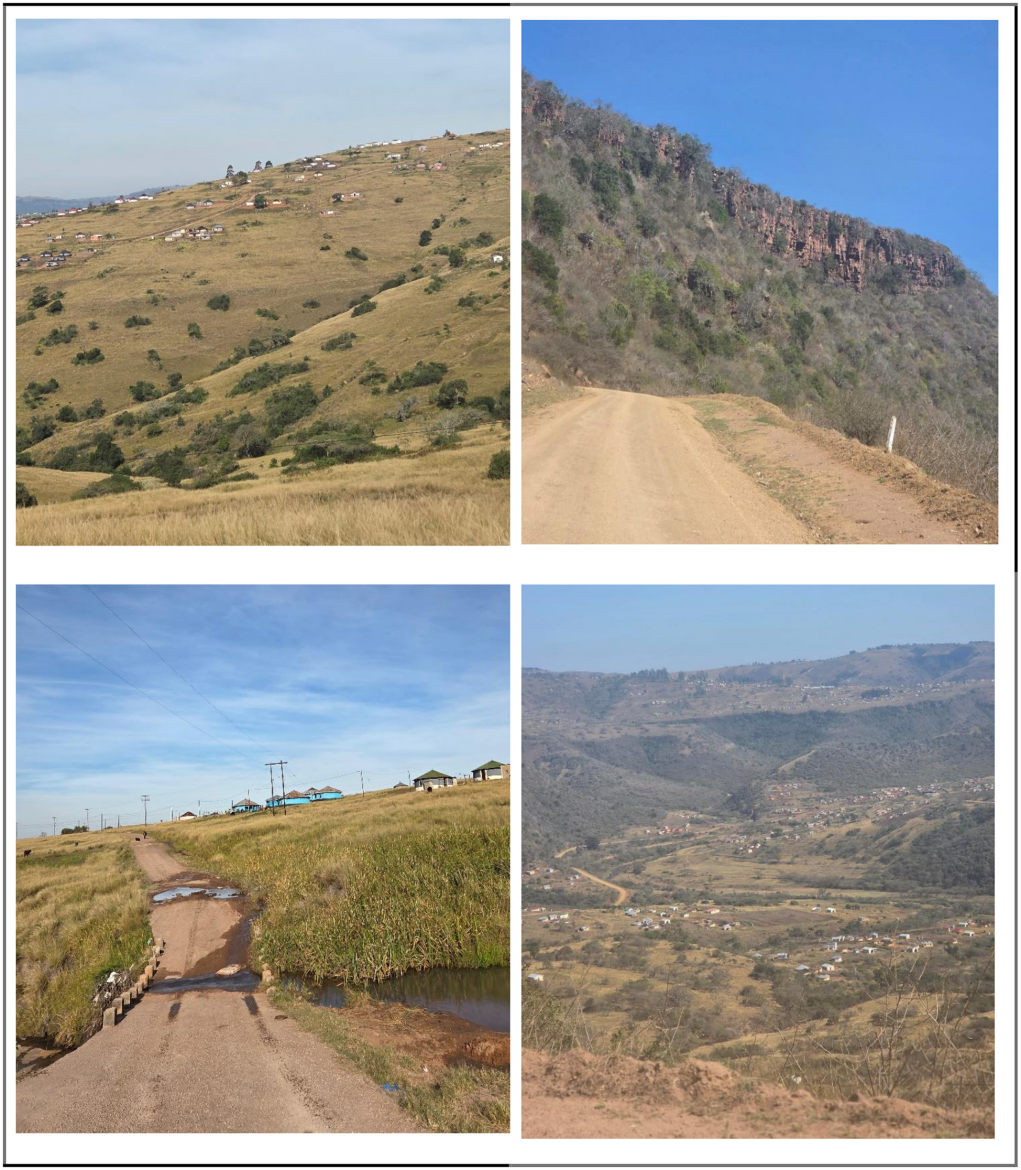
Mt Elias. Reproduced from Duri and Takawira (2026).

Mt Elias is characterized by challenging terrain that significantly impacts the daily travel experiences of learners. Many households are dispersed throughout the landscape, particularly in the valley, where accessibility to schools is often hindered by long distances and uneven surfaces. The geographical features of Mt Elias contribute to a range of difficulties for students navigating their journey to and from school. The valley's steep inclines and poorly maintained paths pose risks to safety, further complicated by the intermittent availability of formal transportation options. Learners living in these remote areas frequently rely on informal travel methods, which can expose them to various hazards, including environmental and social risks. To illustrate the spatial distribution of households in this community, Figure 1 highlights Mt Elias, emphasizing the geographic terrain. Understanding the unique context of Mt Elias is critical for addressing the challenges learners face in accessing school. Mt Elias was selected because its valley-bound terrain, dispersed settlement pattern, and limited transport infrastructure reflect a rural configuration common in parts of KwaZulu-Natal and other provinces. This makes it a relevant case for understanding how topography and infrastructural fragility shape learners' daily access to education.

### 3.2 Research design and methodology

This study adopted a qualitative, exploratory research design to gain in-depth understanding of the school travel experiences of learners in the rural community of Mt Elias, KwaZulu-Natal. The qualitative approach was suitable for capturing the detailed, lived experiences of learners, allowing for the exploration of personal perceptions, challenges, and daily realities that are often overlooked in quantitative studies. A total of 23 learners, drawn from Grades 6 and 7, participated in the study. These learners were purposefully selected to ensure representation of those most affected by long-distance school travel. The data were collected through interviews, which allowed for consistency across participants while also providing flexibility to probe further based on individual responses. The interview questions were carefully developed and phrased in simple, age-appropriate language to ensure clarity and avoid confusion, enabling learners to respond comfortably and meaningfully.

All interviews were conducted in a safe and supportive environment, with informed consent obtained from parents or guardians, and assent from the learners themselves. In some cases, two teachers were present during the interviews to provide emotional support and to act as responsible adults in accordance with school policies. The presence of teachers during the interviews helped create a safe and reassuring environment for learners. However, teachers or parents did not intervene in the interviews or influence the responses provided by the participants. To minimize the influence of the presence of teachers or parents, the researcher explained to learners that there were no right or wrong answers. Learners were assured that their responses would remain confidential and would not affect their academic standing. Questions were framed neutrally and focused on daily experiences rather than evaluating judgments about teachers or school management. The lead researcher is a researcher with experience in rural learner mobility research. Reflexivity was maintained throughout the study by acknowledging how the researcher's professional background could shape probing and interpretation, and by actively prioritizing learners' own meanings and language during interviews and analysis.

The data were analyzed using thematic analysis following the six-phase procedure outlined by Braun and Clarke (2006). Thematic analysis is an effective method for finding, understanding, and describing patterns within qualitative data, highlighting how researchers actively shape the meaning throughout the process. All interviews were audio-recorded, transcribed verbatim, and imported into ATLAS.ti for organization and coding. Analysis began with repeated readings of the transcripts to achieve deep familiarization with learners' narratives, after which inductive coding was undertaken to derive codes directly from the data, focusing on elements such as travel time, environmental risks, emotional responses, transport access, and school engagement. These codes were then clustered into conceptually coherent categories, for instance grouping “bushy areas,” “graveyards,” and “isolated routes” under environmental risk. Themes were iteratively reviewed against the full dataset to ensure internal coherence and distinction, with overlapping or redundant codes refined. Final themes were defined and named to clearly articulate their central organizing concepts, and the write-up interpreted these themes through a transport equity lens to connect empirical patterns with theoretical constructs. The lead researcher conducted the core analysis, supported by regular peer debriefing sessions with the co-author to discuss coding decisions and thematic interpretations, thereby enhancing analytical rigor and limiting potential individual bias. To enhance trustworthiness, an audit trail was maintained through documented coding notes, theme development memos, and iterative revisions within ATLAS.ti. Themes were repeatedly checked against the full dataset to ensure internal coherence and that interpretations were grounded in learners' accounts.

Ethical approval was obtained from the Department of Transport and Supply Chain Management Research Ethics Committee of the University of Johannesburg prior to data collection. Ethical considerations, including anonymity and confidentiality, were strictly observed throughout the research process. The collected data were analyzed using thematic analysis, supported using ATLAS.ti software. ATLAS.ti allowed for systematic coding of the interview transcripts, identification of recurring themes, and interpretation of patterns across the learners' narratives. The use of ATLAS.ti enhanced the rigor and transparency of the analysis by organizing data into clearly defined thematic categories that align with the study's research questions.

## 4 Results

The findings of this study revealed an interplay of factors shaping learners' daily educational experiences in rural Mt Elias. Four key themes that emerged from the data are “Daily schedule,” which highlighted the structured yet constrained routines learners follow; “Transport and accessibility,” which highlighted the logistical challenges and long travel times often required to reach school; “Environmental conditions,” pointing to physical and social surroundings that influence learners' comfort and safety; and “School Engagement,” which captured patterns of attendance, emotional responses to schooling, and the impact of external barriers on learners' participation. Table 1 below summarizes that codes that make up the themes.

**Table 1:** Themes and codes.

**Theme**	**Codes**
Daily schedule	1 h, 30 min, 40 min, 45 min, 4.30 am, 5 am, 5.30 am, 6 am, wake-up time, travel time, late arrival
Transport and accessibility	Mode of transport, walk, minibus taxi, van, cars, scholar transport, lack of transport, transport needs, affordability issues
Environmental conditions	Bushy areas, busy roads, graveyards, isolated areas, muddy roads, rainy season, road conditions, road safety, slippery, tarred roads, path to school, animals, fear of environment
School engagement	School attendance, miss school, school outside community, tiredness, improvement

### 4.1 Daily schedule

Many of the students reported waking up as early as 4:30 or 5:00 am to begin their school day. The lengthy commutes learners make to get to school often on foot or by unreliable transportation make this early wake-up time necessary. The codes 4.30 am, 5 am, 5.30 am, and 6 am demonstrate how much the demands of travel influence learners' everyday schedules. The early mornings, which reflect the physical distance between learners' homes and schools, are a structural requirement rather than a choice. Before the school day starts, the stress of rising early, frequently in cold or harsh weather, adds to emotional and physical tiredness. Travel time emerged as a factor shaping daily experiences of learners.

One learner explained:

“I wake up at 4.30 because I must leave before it gets too late. If I leave at 6:30, I will be late.” (Learner 7)

Another stated:

“Sometimes I am still sleepy when I arrive at school because I walk for almost one hour.” (Learner 12)

These accounts illustrate how travel time shapes the temporal organization of learners' lives. Early rising was not framed as a choice but as a necessity imposed by distance and terrain. The extended commute reduces time available for rest and homework, contributing to fatigue that carries into the classroom.

Late arrivals are particularly associated with the seasonal rainfall. During rainy season, students who live far from school tend to arrive late due to heavy rains and poor road conditions. Mt. Elias experiences significant rainfall during the summer, which disrupts learners' access to education. Additionally, those who need to cross rivers may miss school because of flooded bridges, which are often poorly constructed (see Figure 1). As Learner 3 noted:

“When it rains, the road is slippery and the river is full. Then I arrive late or I don't come.”

Rather than isolated incidents, these narratives reflect a structural pattern in which educational participation is contingent on environmental and spatial conditions.

### 4.2 Transport and accessibility

The most common mode of transport used by learners in Mt Elias was walking. Although a small number of learners reported using taxis, the overwhelming majority relied on walking as their primary mode of transport. When asked how they usually travel to school, one learner responded simply:

“Walk” (Learner 20)

Walking long distances is the norm rather than the exception. The reliance on walking is often due to the lack of transport in the area, compounded by the absence of formal infrastructure such as school buses or scheduled public transport routes. The physical toll of walking several kilometers each day, often in unsafe or uncomfortable conditions, cannot be overstated for some learner. It should be noted that these difficulties have a lesser impact on learners who live close to educational facilities.

Affordability complicates transport access of learners who live far from schools. Affordability reflect the economic constraints faced by many families, who are unable to consistently afford private transport. Mt Elias experiences high unemployment, and many families rely on social grants. For most, paying for private scholar transport may not be financially feasible. A learner explained:

“We do not afford private scholar transport.” (Learner 6)

This quote demonstrates economic exclusion, not mere absence of service. Transport availability does not equate to accessibility. Only a few learners utilize organized minibus taxis service, primarily those attending schools outside of Mt Elias. Learners whose parents can afford good schools typically rely on an organized scholar transport service to take them to schools in nearby town. In contrast, those who live deep in the valley of Mt. Elias often depend on an unsafe transport service known as “vans.” Vans in the study context are converted light delivery vehicles that carry both passengers and goods.

Although scholar transport was mentioned by some learners, its availability and reliability were inconsistent during rainy season. In some cases, learners reported that scholar transport was only available to those living beyond a certain distance threshold, leaving others to fend for themselves. Most learners expressed the need for scholar transport. Scholar transport need captures the widespread desire for a more structured and equitable transport system in Mt Elias. The findings highlight the urgent need for policy reforms that prioritize rural mobility and recognize transport as a fundamental component of educational access.

### 4.3 Environmental conditions

The physical environment through which some learners travel to school presents challenges that significantly impact their safety and well-being. Learners described school routes that traverse bushy areas, isolated paths, and graveyards, which generated fear and vulnerability.

One learner explained:

“We pass near the graveyard and sometimes there are no people. I get scared.” (Learner 9)

Another learner described encounters with animals:

“There are dogs and sometimes snakes in the bush. When we see them, we run.” (Learner 14)

A female learner described her vulnerability. For example:

“As a girl, I often feel unsafe walking alone in the morning, especially if my friends leave me behind.” (Learner 23)

These accounts reveal that the journey to school is not merely physically demanding but emotionally taxing. Fear was not abstract; it was tied to specific locations and recurring experiences. During the rainy season, these risks intensified:

“When it rains, the road becomes muddy. The van also does not come.” (Learner 5)

Such accounts demonstrate how geography, infrastructure, and weather interact to shape learners' sense of safety and dignity.

### 4.4 School engagement

The daily routine, transport difficulties, and environmental risks all have a significant impact on students‘ emotional health and academic engagement. Tiredness was a recurring code in learners' narratives, with many reporting that they arrived at school physically exhausted and mentally unprepared to learn. The fatigue experienced by some learners affects concentration, participation, and overall academic performance. In some cases, learners reported falling asleep in class or struggling to complete assignments due to the energy expended during their commute. For example:

“When I arrive at school, I am tired and I don't concentrate.” (Learner 2)

Another learner described how fatigue affected classroom alertness:

“Sometimes I feel tired in class because I have to walk a long distance.” (Learner 22)

Irregular attendance during the rainy season is a concern. For example, learner 16 and many other learners explained:

“I wait till the rain stops, then I go to school. Sometimes I do not go to school if it is raining too much. The roads are too muddy and slippery.” (Learner 1)

For learners attending schools outside their community, these challenges are even more pronounced due to the added distance. These interruptions not only impede students' academic development but also lessen their sense of continuity and belonging in the classroom. Notwithstanding these challenges, some learners have stated a wish to see improvements made to the educational system as well as to their own circumstances. As learners expressed the need for scholar transport, it shows resiliency and hope among learners in Mt Elias. Therefore, it is critical to address school travel's structural and psychological components. It highlights the necessity of comprehensive interventions that help learners not just get to school but also perform well in school.

## 5 Discussion

The discussion of findings is according to the sub-questions of this study and interprets them using the transport equity framework. The results indicate how access to education in Mt Elias is shaped by spatial, infrastructural, economic, and procedural injustices that intersect to exclude rural learners from safe and reliable school transport systems.

### 5.1 How do learners travel to and from school in Mt Elias?

The findings show that walking is the dominant mode of travel for most learners in Mt Elias, often involving long distances of 30 min to over an hour each way. This is consistent with findings from other rural settings in South Africa and other African countries, where walking remains the default due to limited transport availability and unaffordable alternatives (Porter et al., 2011; Ngidi and Essack, 2022). As of 2019, the main mode of transportation for learners in rural South Africa is walking, with 62.1% of learners making the journey from their homes to school on foot each day (Statistics South Africa, 2020). The learners' journeys frequently start before dawn with some as early as 4:30 a.m. This highlights the disproportionate burden rural learners carry to access education. Distributive injustice was reflected in the uneven provision of scholar transport and the fact that many learners walk for 30 min to over an hour, often starting before dawn, while only a few reported access to taxis or organized transport.

Only a few learners had access to motorized transport such as minibus taxis or van (light delivery vehicles), and even fewer benefitted from formal scholar transport. This aligns with previous research showing that informal and unregulated transport options often fill the gap where formal systems are absent (Cervero and Golub, 2007; Manyaka and Mathebula, 2020). However, informal transport modes are frequently unsafe and unreliable, (Cervero and Golub, 2007) especially during adverse weather, which further undermines punctuality and attendance. From a transport equity perspective, this finding reflects a failure of distributive justice. The benefits of formal transport systems are not reaching the learners who need them most (Pereira and Karner, 2021). The lack of reliable and affordable transport compromises learners' right to education as guaranteed by the Constitution of South Africa (1996), and represents a structural barrier to life opportunities. Moreover, procedural injustice is evident in how community voices, particularly those of learners are often excluded from transport policy discussions that directly affect their lives.

The findings highlight the importance of transport equity in understanding the mobility challenges learners face in Mt Elias. The dominance of long-distance walking, which often begins before sunrise, highlights a clear distributive injustice, where the benefits of formal transport provision fail to reach those who experience the greatest mobility burdens. According to Pereira and Karner (2021), transport equity requires mobility systems to guarantee fair and sufficient access to essential life opportunities. The lack of reliable, safe, and affordable school transport in Mt Elias, therefore, reflects a structural failure to meet even the minimum accessibility thresholds needed for young people to participate fully in education. Furthermore, reliance on unsafe and irregular informal transport modes reflects deeper recognitional and procedural injustices: rural learners' lived experiences and mobility needs are insufficiently acknowledged in planning processes, and their voices remain largely excluded from the decision-making structures that shape school transport provision. From a mobility justice perspective, these inequities are not accidental but rooted in longstanding spatial and infrastructural disadvantages that systematically expose rural learners to heightened risk and fatigue before they even reach the classroom (Bierbaum et al., 2021). The travel patterns identified in Mt Elias demonstrate that transport inequities directly affect educational access, making the importance of assessing rural school transport as both a logistical concern and an equity issue.

### 5.2 What challenges do learners face during their daily travel to school in Mt Elias?

The challenges identified by learners are multifaceted, involving physical, economic, environmental, and emotional hardships. Some learners described walking through bushy paths, isolated areas, and graveyards, all of which evoke fear and discomfort. This resonates with findings by Ngidi and Essack (2022) who reported that rural learners face dangerous social geographies on their school routes. The poor road conditions, and muddy or slippery paths during the rainy season further exacerbates the risks. Learners face inconsistent access to scholar transport, with eligibility often determined by rigid distance criteria that exclude many in need. These challenges reflect systemic issues in KwaZulu-Natal's scholar transport policy, where administrative hurdles and limited funding result in fragmented service delivery (Manyaka and Mathebula, 2020; Government Technical Advisory Centre, 2021).

Economic barriers were a significant issue as well. Many learners were unable to afford informal transport, even when it was available. This situation highlights the lack of affordability and transport options. Affordability and transport options are key concerns in the egalitarian aspect of transport equity, which focuses on reducing disparities in access and minimizing exposure to risk (Pereira and Karner, 2021; Bierbaum et al., 2021). Gendered experiences were also noted with some female learners expressing safety concerns during early-morning walks, especially when passing through isolated areas. This finding is in line with previous studies that show rural school travel often exposes girls to gender-based vulnerabilities, including harassment and fear (Ngidi and Essack, 2022). From a recognitional justice standpoint, the risks that learners face point to the need for gender-sensitive planning that acknowledges and responds to the unique needs of different learner groups (Eagle and Kwele, 2021). Recognitional injustice emerged in how learners' lived realities, including fear when passing graveyards, walking through bushy and isolated routes, and gendered vulnerability during early-morning travels.

The combined effects of barriers such as fatigue, tardiness, emotional stress, and safety concerns can greatly impede learners' engagement in school and their academic achievements. These challenges extend beyond daily routines; they influence the entire learning experience and ultimately shape overall performance. Some learners reported falling asleep in class or missing lessons, and these findings mirror previous research that explored sleeplessness in class and commuting (Pradhan and Sinha, 2017; Pem et al., 2023). The findings reveal that transport-related inequalities in rural Mt. Elias lead to unequal exposure to danger, limited access to mobility, and minimal influence in policy-making. These disparities highlight critical issues in the transport equity framework. The voices of learners are often overlooked in transport policy discussions, resulting in solutions that do not effectively address their unique needs. Addressing the challenges that learners in rural areas face requires more than just infrastructure; it demands a holistic and equity-driven approach that centers on the lived realities of these rural learners and integrates their voices into policy and planning processes.

The challenges experienced by learners in Mt Elias reveal deep transport inequities that are structural rather than incidental, aligning closely with the concerns raised by transport equity and mobility justice framework. The physical dangers, emotional distress, and economic barriers learners face illustrate egalitarian failures, where certain groups are disproportionately exposed to risk and deprivation despite having the same fundamental right to education. Transport equity requires reducing disparities in access and minimizing exposure to harm (Pereira and Karner, 2021). Yet the everyday realities in Mt Elias are associated with unsafe walking routes, unaffordable transport, and gender-specific vulnerabilities. These mobility needs are far from being met. The experiences described by learners also reflect a breakdown in recognitional justice, particularly where gendered safety concerns and local travel realities are not incorporated into planning or policy responses. From a mobility justice perspective, these inequities are rooted in broader structural conditions, where limited transport options and fragmented scholar transport systems reflect longstanding patterns of uneven investment and exclusion from decision-making (Bierbaum et al., 2021). The results indicate that transport challenges in Mt Elias extend beyond logistical concerns and reflect underlying systemic inequities. Addressing the transport challenges requires placing the lived experiences of rural learners in policy and planning processes, ensuring that their needs and voices shape more just, inclusive mobility systems.

## 6 Limitations

While this study provides valuable insights into the school travel experiences of rural learners in Mt Elias, there are some limitations that should be acknowledged. The research relied on purposive sample of 23 Grade 6 and 7 learners, which limits the generalizability of the findings. Although qualitative research is not intended to produce statistically representative results, the experiences shared may not fully reflect the diversity of travel challenges faced by learners in other grades or rural communities beyond Mt Elias. Future research could extend this work by incorporating mixed-method approaches and comparative multi-site analysis to examine how transport inequities vary across seasons and educational stages.

The study was conducted within a single rural area. Rural communities in South Africa are heterogeneous, differing in terrain, settlement density, transport infrastructure, and policy implementation. As such, the findings are not generalizable to all rural contexts. Instead, the study offers analytic insights into how terrain, infrastructure fragility, and policy thresholds interact in valley-bound settlements. Transferability, therefore, relates to contexts with similar topographic and infrastructural characteristics.

The study applied a transport equity framework, it did not empirically measure equity outcomes using quantitative indicators such as travel time variability, cost burdens, or comparative access levels. Future studies could incorporate mixed methods to deepen and triangulate the findings. Although teachers and parents did not participate in the discussions, their presence may have influenced how freely some learners expressed concerns, particularly regarding school-related issues.

## 7 Recommendations

Based on the findings of this study, several recommendations are proposed to improve school transport access and learner well-being in rural communities like Mt Elias:

The Department of Transport, in collaboration with the Department of Basic Education, should extend scholar transport provision to include learners in remote and topographically challenging areas even if they fall just short of existing distance criteria. A more flexible, eligibility model based on need would ensure that vulnerable learners are not excluded from support due to rigid distance thresholds

For learners who live in remote valleys or far from school, the provision of powered bicycles or e-bikes offers a cost-effective and environmentally friendly alternative to walking long distances. The introduction of powered bicycles is proposed cautiously as a pilot-level, exploratory intervention rather than a central policy solution. This form of active transport reduces travel time, minimizes physical fatigue, and improves punctuality and attendance. Pilot programmes could be implemented in collaboration with NGOs, private partners, or corporate social responsibility initiatives, especially in areas where road conditions do not permit school buses. Similar initiatives in rural India and parts of Africa have shown success in improving school attendance and reducing dropout rates. For powered bicycle schemes to succeed, maintenance support, safety training, and storage facilities should be provided alongside the bicycles.

## 8 Conclusion

This study aimed to address the questions: (1) What are the daily school travel experiences of learners in the rural community of Mt Elias, KwaZulu-Natal? (2) What challenges do learners face during their daily travel to school in Mt Elias? Through qualitative inquiry, the study examined how learners travel to and from school and the challenges they face, revealing how access barriers hinder equitable access to education. From a transport equity perspective, the study highlighted critical issues of distributive injustice in the uneven allocation of transport services; recognitional injustice in the limited attention to local realities and gendered risks; and procedural injustice in the exclusion of learners and caregivers from transport planning. Addressing these layered inequalities requires not only infrastructure investment, but also responsive, participatory, and context-sensitive policies that center the voices of rural communities.

Although previous South African studies have documented long walking distances, unsafe routes, and transport-related absenteeism in rural contexts, this study makes two distinct contributions. Much of the existing literature treats rural areas as relatively uniform. This study demonstrates that intra-rural variation matters. The study contributes a terrain-sensitive understanding of rural mobility inequity, highlighting how geographic micro-context shapes educational access. The study foregrounds learners as primary narrators of their mobility experiences. In many transport and education studies, children are treated as statistical categories rather than as informants. The voices of learners in this study expand the conversation beyond physical access to dignified, psychologically safe mobility.

This research offers a context-specific contribution that addresses the micro-level variations within rural schooling environments and highlights how localized geography and governance shape educational access and equity. By identifying the mobility challenges faced by learners in Mt Elias, the study contributes to future policy development, such as increasing the availability of subsidized learner transport, improving infrastructure, and implementing safety measures. The study also supports a number of the Sustainable Development Goals (SDGs) of the UN, especially SDG 4 (Quality Education), which aims to promote inclusive and equal access to education by identifying structural and logistical constraints. By highlighting the fact that access to education is only completely achieved when it is ensured, secure, and equal, this study advances our knowledge of the relationship between education, transportation, and rural development.

## Data Availability

Due to the sensitive nature of data, the raw data are not publicly available. Anonymized excerpts may be provided upon reasonable request.
